# An international parentage and identification panel for the domestic cat (*Felis catus*)

**DOI:** 10.1111/j.1365-2052.2007.01632.x

**Published:** 2007-08-01

**Authors:** M J Lipinski, Y Amigues, M Blasi, T E Broad, C Cherbonnel, G J Cho, S Corley, P Daftari, D R Delattre, S Dileanis, J M Flynn, D Grattapaglia, A Guthrie, C Harper, P L Karttunen, H Kimura, G M Lewis, M Longeri, J-C Meriaux, M Morita, R C Morrin-O'Donnell, T Niini, N C Pedersen, G Perrotta, M Polli, S Rittler, R Schubbert, M G Strillacci, H Van Haeringen, W Van Haeringen, L A Lyons

**Affiliations:** * Department of Population Health and Reproduction, School of Veterinary Medicine, University of California Davis Davis, CA 95616, USA; † Labogena, INRA, Domaine de Vilvert 78352, Jouy en Josas Cedex, France; ‡ L.G.S. Laboratorio di Genetica e Servizi Via Bergamo 292, 26100 Cremona, Italy; § Australian Equine Genetics Research Centre, University of Queensland QLD, Australia; ¶ GENINDEXE 6 Rue des Sports, 17000 La Rochelle, France; ** Laboratory of Equine Genetics, Korea Racing Association Gyonggi-do, Korea; †† Veterinary Genetics Laboratory, School of Veterinary Medicine, University of California Davis Davis, CA 95616, USA; ‡‡ 7 ANTAGENE SA, F-69760 Limonest France; §§ Weatherbys DNA Laboratory, C/O Irish Equine Centre, Johnstown, Naas, Co Kildare Ireland; ¶¶ Laboratório Hereditas, Brasilia, DF and Graduate Program in Genomic Sciences, Catholic University of Brasilia DF, Brazil; *** Veterinary Genetics Laboratory, Faculty of Veterinary Science, University of Pretoria Onderstepoort, South Africa; ††† Faba Jalostus, PL 40, 01301 VANTAA, Urheilutie 6, Vantaa Finland; ‡‡‡ Section of Parentage Verification, Division of Animal Genetics, Maebashi Institute of Animal Science, Livestock Improvement Association of Japan, Inc. Maebashi Gunma, Japan; §§§ Faculty of Veterinary Medicine, Istituto di Zootecnica, University of Milan Milan, Italy; ¶¶¶ Oy Triniini Company PO Box 36, FIN-00501, Helsinki, Finland; **** Eurofins Medigenomix Fraunhofer Strasse 22, 82152 Planegg, Germany; †††† VHL, Agro Business Park 1006708 PW Wageningen, the Netherlands

**Keywords:** cat, feline, identification, microsatellite, parentage

## Abstract

Seventeen commercial and research laboratories participated in two comparison tests under the auspices of the International Society for Animal Genetics to develop an internationally tested, microsatellite-based parentage and identification panel for the domestic cat (*Felis catus*). Genetic marker selection was based on the polymorphism information content and allele ranges from seven random-bred populations (*n* = 261) from the USA, Europe and Brazil and eight breeds (*n* = 200) from the USA. Nineteen microsatellite markers were included in the comparison test and genotyped across the samples. Based on robustness and efficiency, nine autosomal microsatellite markers were ultimately selected as a single multiplex ‘core’ panel for cat identification and parentage testing. Most markers contained dinucleotide repeats. In addition to the autosomal markers, the panel included two gender-specific markers, *amelogenin* and *zinc-finger XY*, which produced genotypes for both the X and Y chromosomes. This international cat parentage and identification panel has a power of exclusion comparable to panels used in other species, ranging from 90.08% to 99.79% across breeds and 99.47% to 99.87% in random-bred cat populations.

## Introduction

DNA-based genetic testing is used for most domesticated animals to confirm identity, to determine parentage and, particularly, to validate registries (Kemp *et al.* 1995; Bowling *et al.* 1997; Nechtelberger *et al.* 2001; DeNise *et al.* 2004). The domestic cat is one of the leading household pets, but parentage and identification testing lags for this species because no cat registry requires parentage validation. DNA-based tests for highly prevalent diseases of cats, such as polycystic kidney disease (Lyons *et al.* 2004) and hypertrophic cardiomyopathy (Meurs *et al.* 2005), and for popular coat colour traits, such as agouti (Eizirik *et al.* 2003), points (Lyons *et al.* 2005b) and brown variants (Lyons *et al.* 2005a), are currently driving DNA profiling rather than pedigree validation.

The vast majority of cats in the world are randomly bred, although interest in fancy breeds has steadily increased. Households in the USA are the most likely to have a cat of a fancy breed; however, the likelihood is low, only 10–15% or less (Louwerens *et al.* 2005). Thirty of 80 major breeds (Morris 1999) are recognized by most cat fancy associations in the world. However, Persians and related breeds, such as Exotics, represent the overwhelming majority. Most cat breeds have been developed by crossing older ‘foundation’ breeds or by hybridizing domestic cats with small wild felid species such as Asian leopard cats, jungle cats and servals (Robinson 1991; Vella *et al.* 1999). Hence, genetic profiling in cats may need to consider the sub-structures of cat populations, including different species. However, sub-structuring and selective sweeps may not be as significant for cats when compared with dog breeds because single-gene traits, not complex traits, define most cat breeds. Additionally, selection in cats has not occurred for nearly as long as in dogs and cat populations across the world tend to be large and freely bred. Therefore, cat microsatellite markers may have more uniform inter-breed allele frequencies than the more genetically isolated, domesticated dog breeds (DeNise *et al.* 2004).

Standardized genetic tests are important for sharing information, combining datasets and assisting with population management. These tests are particularly important for purebreds, especially when individuals transfer between registries and countries. The scientific community provides oversight of industry standards pertaining to parentage and identification panels. Peer-review, research collaborations and forums and comparison tests hosted by the International Society for Animal Genetics (ISAG) allow both formal and informal oversight. We describe herein the results of an ISAG comparison study for cats using 461 cats genotyped for 19 microsatellites by 17 worldwide commercial and research laboratories.

## Materials and methods

### Animals

The microsatellite marker analysis included 15 cat populations primarily from the USA (Table 1). For the cats of a particular breed, pedigree information determined that the cats did not have grandparents in common. Seven feral and random-bred cat populations were collected from different regions in the USA, Europe and Brazil (Table 1). Kinship of the random-bred cats was minimized by avoiding obvious parent–offspring combinations. Microsatellites were sequenced from several homozygous cats (from the Persian and Korat breeds and the Hawaii and Texas random-bred populations) to determine the repeat lengths of the alleles.

**Table 1:** Cat breeds and populations used to identify parentage panel markers.^1^

Cat population	No.	Mean alleles	Allele range	Mean He^2^	Mean Ho^3^	Mean PIC^4^
Davis, CA	25	4.2	1–8	0.52	0.45	0.59
Ithaca, NY	41	7.0	3–11	0.68	0.58	0.64
Caldwell, TX	31	6.7	3–9	0.69	0.61	0.65
Maui, HI	63	7.0	3–10	0.63	0.55	0.60
Brazil	28	6.2	2–10	0.68	0.64	0.64
Finland	42	6.4	2–10	0.65	0.60	0.62
Italy	31	7.8	3–12	0.73	0.68	0.69
Abyssinian	15	3.0	1–5	0.44	0.42	0.38
Birman	33	3.3	1–6	0.41	0.36	0.35
Burmese	17	3.5	1–6	0.49	0.36	0.45
Havana	13	3.2	2–6	0.44	0.42	0.40
Maine Coon	26	4.5	2–6	0.56	0.44	0.52
Persian	36	5.3	2–8	0.60	0.49	0.56
Siamese	36	4.0	2–7	0.48	0.41	0.43
Siberian	24	6.1	2–9	0.70	0.69	0.66
All random	261	6.5	1–12	0.65	0.59	0.63
All breeds	200	4.3	1–9	0.51	0.45	0.47
Total	461	5.2	1–12	0.58	0.51	0.55

1 Data were determined for 19 microsatellite markers that were analysed in the comparison tests.

2 Mean expected heterozygosity.

3 Mean observed heterozygosity.

4 Polymorphism information content.

### Comparison tests

For the 2004 ISAG Cat Comparison Test, fluorescently labelled aliquots of primers (Applied Biosystems), DNA samples (from 23 cats) and PCR protocols were shipped to 20 laboratories interested in performing the comparison test. The cat samples included (i) two buccal swabs from each of eight cats that formed a small, inbred pedigree, (ii) two buccal swabs from each of 11 random-bred cats and (iii) three controls, including two buccal swabs and one tissue-derived DNA sample. Allele sizes of the three control cats were provided prior to the submission of results (Table 2) and were determined by the two UC Davis laboratories using both gel-based (ABI 377 DNA Analyzer, Applied Biosystems) and capillary-based (ABI 3730, Applied Biosystems) systems. The participating laboratories were expected to amplify all markers in all the cats to assess (i) the efficiency of marker amplification, (ii) the ease of use in multiplex, (iii) the ease of genotyping, (iv) the accuracy in allele determination, (v) the consistency across genotyping instrumentation and allele-calling software, (vi) the consistency of genotypes between DNA isolated from buccal swabs and other sources, (vii) the ability to determine gender and (viii) the ability to resolve parentage. A genotype was considered an error if it did not correspond to the consensus sizes obtained across the laboratories. The UC Davis laboratory (L.A. Lyons) distributed the samples and marker information and compiled and analysed the results.

**Table 2:** Allele sizes for control cat DNA samples.

		Control sample alleles (bp)^1^			
		
Marker	Forward primer 5′–3′; Reverse primer 5′–3′	Fcat-4406	Fcat-4649	Fcat-4444	CCL-94^2^
*FCA069*	AATCACTCATGCACGAATGC; AATTTAACGTTAGGCTTTTTGCC	110/110	106/108	108/112	107/109
*FCA075*	ATGCTAATCAGTGGCATTTGG; GAACAAAAATTCCAGACGTGC	140/140	140/140	134/136	136/136
*FCA105*	TTGACCCTCATACCTTCTTTGG; TGGGAGAATAAATTTGCAAAGC	199/199	191/193	191/193	193/193
*FCA149*	CCTATCAAAGTTCTCACCAAATCA; GTCTCACCATGTGTGGGATG	130/132	124/132	124/128	128/128
*FCA220*	CGATGGAAATTGTATCCATGG; GAATGAAGGCAGTCACAAACTG	216/216	216/218	214/216	214/216
*FCA229*	CAAACTGACAAGCTTAGAGGGC; GCAGAAGTCCAATCTCAAAGTC	164/168	170/170	166/170	168/168
*FCA310*	TTAATTGTATCCCAAGTGGTCA; TAATGCTGCAATGTAGGGCA	124/126	136/136	136/138	120/124
*FCA441*	ATCGGTAGGTAGGTAGATATAG; GCTTGCTTCAAAATTTTCAC	161/165	161/165	165/169	159/159
*FCA678*	TCCCTCAGCAATCTCCAGAA; GAGGGAGCTAGCTGAAATTGTT	232/232	224/232	232/232	204/210

1 Allele sizes were determined on an ABI 3730 DNA Analyzer (Applied Biosystems).

2 ATCC cat cell line CCL-94 (ATCC).

The 2006 ISAG Cat Comparison Test had the same goals and evaluated the same 19 microsatellite markers as well as two gender-specific markers, *amelogenin* (*AMEL*) and *zinc-finger XY* (*ZFXY*) (Pilgrim *et al.* 2005), and 22 cat DNA samples, including one cell line from ATCC (CCL-94). Twenty-one laboratories requested the feline comparison test reagents and information. For standardization, the Veterinary Genetics Laboratory in South Africa provided reference genotypes for two markers per cat. The Van Haeringen Laboratory in the Netherlands served as the data analysis laboratory.

## Results

Seven random-bred populations (containing 261 cats) and eight common breeds (containing 200 cats) were used to evaluate 19 microsatellite markers for inclusion in the Cat Comparison Test (Table 1). The mean number of alleles for all markers in the breeds was 4.3 (3.0–6.1); in the random-bred cat populations, it was 6.5 (4.2–7.8). The mean PIC was 0.47 (0.35–0.66) in the breeds and 0.63 (0.59–0.69) in the random-bred cats. None of the autosomal markers had a significant departure from Hardy–Weinberg equilibrium nor had a significant increase of homozygote genotypes. The powers of exclusion (PE) ranged from 90.1% to 99.8% across the purebreds, with the Siberian having the highest PE for a majority of the markers. No specific breed had the lowest PE for all the markers. The Birman breed had the lowest combined PE of 90.08%. The PE for the seven groups of random-bred cat were similar, ranging from 99.5% to 99.9%.

### 2004 ISAG Cat Comparison Test

The 2004 Cat Comparison Test consisted of 4940 potential genotypes derived from 20 non-control cats, 19 markers and 13 reporting laboratories. The range of discrepancies, when compared with the consensus sizes obtained by a majority of laboratories for all markers, was 1–40 genotyping errors. The error rate was approximately 4.13% across all markers, as calculated from 130 discrepancies and 74 non-reported values. One laboratory, which reported data from an ABI 310 instrument, had significantly different results. The error rate dropped to 3.55% after discarding results from this laboratory. Most genotyping discrepancies occurred in the random-bred cats, which did not have related cats for comparison.

*FCA649* had the highest error rate and was the most difficult to consistently amplify. Single-base-pair mutations, detected only on an ABI 3700 DNA Analyzer, were identified for marker *FCA097*. Null alleles were identified for marker *FCA453* and this marker had inconsistent amplification. Markers *FCA149* and *FCA097* had low quantities of amplification products. *FCA220* was reported to have low amplification for one allele, but no errors were reported. Marker *FCA651* was not highly informative. Markers *FCA005*, *FCA026*, *FCA069*, *FCA075*, *FCA097*, *FCA201*, *FCA229* and *FCA293* were polymorphic and produced robust amplification products in several wild felid species, including lions (*n* = 4), cheetahs (*n* = 5) and Black-footed cats (*n* = 14). Markers *FCA026* and *FCA069* had null alleles in Asian leopard cat (*n* = 6) and serval cat hybrids (*n* = 10).

### 2006 ISAG Cat Comparison Test

Participating laboratories had the potential of generating 9186 data points. Some laboratories genotyped only the markers that were suggested as a core panel from the previous comparison test or did not type the cell line. Therefore, the actual total dataset was 8104 data comparisons. Eighty-nine per cent (7221 genotypes) of the data points were consistent across a majority of the laboratories. Fifty-six of the data points were not reported and were considered errors. Only two of the participating laboratories reported results from the gender-specific markers and only two samples were gender-discordant.

For nine markers, 96–98% of the data were called consistently and six of these nine loci were selected for the core panel. The single tetranucleotide marker *FCA441*, which was evaluated because it overlapped with forensic markers, had low consistency at 75%. However, two of the 11 laboratories did not convert their genotypes to the allele sizes of the provided standards; thus the accuracy of the data could not be determined. For *FCA105*, data from one of the 11 reporting laboratories were not converted to the standards, so these data were also discarded. Eliminating these discrepancies, a majority of markers had over 90% accuracy in data consistency.

Nine microsatellite markers with the lowest error rates and the most consistent PCR product amplifications were ultimately selected for the core parentage and identification panel (Tables 3 and 4). The X-linked markers *FCA240* and *FCA651* were replaced with the gender-specific markers *AMEL*, which produces a 194-bp Y allele and a 214-bp X allele, and *ZFXY*, which produces a 163-bp Y allele and a 166-bp X allele.

**Table 3:** Population data for genetic markers in the cat parentage and identification panel.

Marker	No. of breeds	No. of random	Allele range (bp)^1^	PIC^2^ breeds	PIC random	He^3^ breeds	He random	Ho^4^ breeds	Ho random
*FCA069*	186	195	88–116	0.77	0.71	0.80	0.74	0.51	0.65
*FCA075*	181	209	112–146	0.73	0.75	0.76	0.78	0.48	0.76
*FCA105*	182	228	173–207	0.72	0.84	0.75	0.86	0.54	0.82
*FCA149*	184	229	120–136	0.79	0.72	0.82	0.75	0.67	0.64
*FCA220*	156	196	208–224	0.37	0.44	0.39	0.46	0.28	0.43
*FCA229*	152	193	150–174	0.56	0.67	0.59	0.71	0.45	0.63
*FCA310*	182	210	112–138	0.66	0.69	0.71	0.73	0.59	0.65
*FCA441*	168	195	133–173	0.73	0.68	0.77	0.72	0.56	0.65
*FCA678*	168	204	222–236	0.59	0.68	0.63	0.72	0.43	0.63

1 All allele sizes were determined on an ABI 3730 DNA Analyzer (Applied Biosystems).

2 Polymorphism information content.

3 Mean expected heterozygosity.

4 Mean observed heterozygosity.

**Table 4:** Genetic marker panel for cat parentage and identification.

					Power of exclusion (PE) (min–max)	
					
Marker	Cat Chr.	Nucleotide repeat	Label	Final primer concentration (*μ*M)^5^	Breeds	Random-bred
*FCA069*	B4	AC	VIC	0.20	0.1324–0.5336	0.3958–0.5948
*FCA075*	E2	TG	NED	0.10	0.1442–0.5771	0.4240–0.5992
*FCA105*	A2	TG	PET	0.20	0.2221–0.5585	0.6110–0.7101
*FCA149*^1^	B1	TG	PET	0.18	0.1783–0.5995	0.3586–0.5767
*FCA220*	F2	CA	FAM	0.30	0.0000–0.3383	0.1851–0.4221
*FCA229*	A1	GT	NED	0.25	0.0452–0.5131	0.3927–0.5813
*FCA310*^1^	C2	(CA)_5_TA(CA)_7_TA(CA)_8_	FAM	0.30	0.1196–0.5256	0.3417–0.5611
*FCA441*^2^	D3	TAGA	VIC	0.15	0.2061–0.5774	0.3388–0.5505
*FCA678*^4^	A1	AC	NED	0.25	0.0415–0.4908	0.3016–0.5715
*AMEL*^3^	XY	—			N/A	N/A
*ZFXY*^3^	XY	—	PET	0.20	N/A	N/A
Total PE					0.9008–0.9979	0.9947–0.9987

1 Markers that are of the first 10 published feline microsatellites (Menotti-Raymond & O'Brien 1995).

2 A marker that is currently included in the feline forensic panel (Menotti-Raymond *et al.* 2005).

3 The two markers on the X and Y chromosomes were added to the panel after the comparison test (Pilgrim *et al.* 2005).

4 Newly designed primers presented herein for *FCA678* generate a product 30 bp less than originally published primers.

5 Forward and reverse primers (Table 2) are used in equal concentrations to make combined concentrations for each marker. Final PCR reaction volumes were 15 *μ*l. The suggested PCR conditions include a 5-min denaturation at 95 °C, followed by 35 cycles of denaturation at 95 °C for 1 min, annealing at 58 °C for 30 s and extension at 72 °C for 30 s, with a final 30-min extension at 72 °C.

For each of the markers in the core panel, the nucleotide length of the most common allele was determined by sequence analyses in different cat breeds (Table 5). The direct comparison of electrophoretic size, repeat unit length and designated alphabetical nomenclature for the cat profiling panel is presented. SNPs were noted in several markers, suggesting that similarly sized alleles are not identical by descent across all populations. SNPs were detected in the unique flanking sequence or within the repeat units in four markers: AF130500:g.167G>C in *FCA069*, AF130546:g.166G>A in *FCA149*, AF130571:g.166A>C in *FCA220* and AF130626: g.67C>T in *FCA441*. Table 5 presents the electrophoretic sizes of the alleles for two instruments (ABI 377 and ABI 3730) and the suggested letter or repeat unit nomenclature conversion.

**Table 5:** Suggested conversion of letter nomenclature to allele size for the cat parentage and identification panel.

	Letter nomenclature for alleles																						
	
Marker	C	D	E	F	G	H	I	J	K	L	M	N	O	P	Q	R	S	T	U	V	W	X	377
*FCA069*					93+4	95	97	99	101	103	105 (21)	107+4	109	111	113	115	117						+5
*FCA075*	104	106	108	110	112	114	116	118	120	122	124 (23)	126	128	130	132+7	134	136	138	140	142	144	146	+4
*FCA105*					173	175	177	179	181	183	185 (16)	187	189	191	193	195	197−5	199	201	203	205	207	−2
*FCA149*					122+1	124	126	128+1	130	132	134 (20)	136	138	140	142								+2
*FCA229*					150	152	154	156	158	160	162 (21)	164	166	168−2	170	172	174	176					0
*FCA310*					112	114	116	118	120	122	124 (15)	126	128	130	132	134	136	138−2	140				−2
*FCA441*				145	147	149	151	153	155+0	157	159 (12)+0	161	163	165	167	169	171	173					+2
*FCA678*						186	188	190	192	194−2	196 (17)	198	200	202	204	206							0
*FCA220*							208	210	212^1^	214^1^	216 (18)	218	220	222	224	226	228						+2

1 A 1-bp insertion has been noted in marker *FCA220*, producing additional alleles L1 (215 bp) and K1 (213 bp).

All allele sizes were determined on an ABI 3730 DNA Analyzer (Applied Biosystems). Primers for marker *FCA678* have been redesigned from the original publication (Menotti-Raymond *et al.* 1999), producing a shorter product. Alleles that are underlined have been sequenced for homozygous individuals from different breeds or populations. The actual nucleotide length can be determined by the addition or subtraction of the noted number of base pairs that is presented alongside the allele. The numbers of repeats in the core unit of the microsatellite are presented in parentheses for the middle (M) allele. The number of repeats was directly determined for the alleles that were sequenced and interpolated for the M allele. The anticipated conversion required for the ABI 377 is presented in the last column as the number of base pairs that need to be added or subtracted when compared with the ABI 3730.

## Discussion

One of the most important aspects of a DNA marker panel for parentage applications is the correct exclusion of non-fathers. The ability to resolve paternity when closely related individuals are tested as alleged fathers is particularly critical in inbred populations. Most microsatellites tested for the panel had comparable variation over all breeds, so the selection of microsatellites was based on other standard criteria, such as small product size, robustness of amplification and clarity in scoring.

Individual identification is also important in forensic applications; however, marker panels developed for forensic purposes ultimately need to be concerned with efficiency (for amplifying trace amounts of DNA and degraded DNA). The core markers in the feline parentage and identification panel appear to be valuable for individual identification purposes. As most of the markers in the proposed panel generate PCR products smaller than those in a recently recommended feline forensic panel (Menotti-Raymond *et al.* 2005), the international cat parentage and identification panel described in this study could also provide a useful complementary tool in forensic applications.

The proposed international cat parentage and identification panel consists of nine microsatellite markers with a cumulative PE of 90.1–99.8% for purebreeds and 99.5–99.9% in random-bred populations. This power is within the range of that estimated for parentage-testing panels of other domestic animal species. However, due to breed sub-structuring, panels in other species generally include more markers and thus are more costly (Bowling *et al.* 1997; Ichikawa *et al.* 2001; Tozaki *et al.* 2001; DeNise *et al.* 2004). One of the newest cat breeds, the Siberian, had variation comparable with a random-bred population. One of the oldest cat breeds, Birmans, are the third most popular cat breed in the Cat Fanciers’ Association (CFA), having approximately 4000 cats registered yearly. If the registered number represents only 25% of the breed, and a cat's life span is about 14 years, then the current Birman population could be approximately 224 000 cats in the USA, with 50% males expected. Thus, a PE of 90.1% may not be sufficient to uniquely identify all individuals in a population of 112 000 Birmans, but may be sufficient to exclude potential sires. Additional markers could improve the PE for particular breeds, especially markers that were highly polymorphic in breeds where a lower overall PE was found exclusively from the nine-marker panel. For example, markers *FCA736*, *F141* (Menotti-Raymond *et al.* 2005), *FCA391* and *FCA090* (Lipinski *et al.*, submitted) had high variation in Birmans. These four markers may be of benefit for paternity exclusion in Birmans and may be suggested as additions to the core panel provided they are robust in as many breeds as possible.

The first publication of microsatellites in the cat included 10 markers (Menotti-Raymond & O'Brien 1995). Several researchers have used most of these 10 markers in population studies that have included wild and domestic cats (Wiseman *et al.* 2000; Beaumont *et al.* 2001; Randi *et al.* 2001). Of these 10 markers, *FCA149* and *FCA310* are included in the core cat parentage and identification panel. Additionally, one marker in the final panel is a tetranucleotide repeat and currently used in a cat forensic panel (Menotti-Raymond *et al.* 2005).

Nomenclature is imperative for the standardization of marker data. Allele sizes varied among instruments, as noted in Table 5. Some markers did not vary, while other markers had up to 6-bp discrepancies. The use of standard DNA controls, such as the ATCC cell line CCL-94 and the establishment of exact nucleotide lengths of marker alleles, allow for proper conversion and data sharing. The identified SNPs in four markers indicate that electrophoretically determined alleles are not always identical by descent.

The correct assignment of gender is also important to support an animal's identification. The two microsatellite markers were replaced by *AMEL* and *ZFXY*, which provide both X- and Y-specific amplicons and more accurate gender determination. The *SRY* locus provides gender determination in the published forensic panel for cats (Menotti-Raymond *et al.* 2005); however, for this marker, females would present the same as a failed PCR reaction, making male identification less accurate.

The international cat parentage and identification panel consists of markers that can be amplified in one reaction. It has sufficient power of exclusion and the markers do not have high mutation rates that would suggest false parental exclusions. The cat panel markers are supported by 17 worldwide laboratories that have different levels of expertise and experience and use a variety of different instrumentation for amplification and genotyping. The robustness of the panel should be further tested with unique and highly inbred populations and the utility of the panel could be expanded by incorporating markers for common diseases or phenotypes.
